# The Strength of Rail Vehicles Transported by a Ferry Considering the Influence of Sea Waves on Its Hull

**DOI:** 10.3390/s24010183

**Published:** 2023-12-28

**Authors:** Alyona Lovska, Juraj Gerlici, Ján Dižo, Vadym Ishchuk

**Affiliations:** Department of Transport and Handling Machines, Faculty of Mechanical Engineering, University of Žilina, Univerzitná 8215/1, 010 26 Žilina, Slovakia; alyona.lovska@fstroj.uniza.sk (A.L.); juraj.gerlici@fstroj.uniza.sk (J.G.); vadym.ishchuk@fstroj.uniza.sk (V.I.)

**Keywords:** train ferry transportation, tensiometers, rail vehicle, container, dynamic load, strength, stability

## Abstract

The article presents the results of a determination of the load attributed to rail vehicles transported by a ferry, considering the influence of sea waves on its hull. A mathematic model describing the displacements of a train ferry, which transported rail vehicles on its decks during rolling oscillations, was created. Calculated accelerations were used to identify the load of components from a dynamics point of view and they were subsequently applied as an input to the analysis of the strength of the open wagon main-bearing structure in a standard scheme of interaction with a train ferry deck. The calculated maximal equivalent stresses in the structure of the fastening units exceeded the valid permissible values. To confirm the theoretical results, experimental studies focused on the strength analysis of the open wagon placed on the railway ferry deck, which was performed in real operational conditions. Electrical voltage sensors were used to determine stress distribution in the areas where the body was attached to the deck. In this case, sensors of the strain gauges, i.e., tensiometers, were used. The base of 25 mm is a dimensional parameter and the resistance, 124 Ohms, is the tensiometer parameter. Verification has been performed and, based on the obtained experimental results, it has been established that the hypothesis’ adequacy is not rejected. The authors developed some measures for adaption of the lashing devices for rail cars on train ferries, which can ensure their safe transportation by sea. The strength calculation demonstrated that, in the new scheme of securing the transported railway vehicles on the railway train ferry, the stresses in its structure do not exceed the permissible values. The article also includes information about the results of the strength calculation of a container placed on a roll trailer transported by a train ferry. This research will contribute to the development of measures regarding the safety of railway vehicle transportation by sea ferry and better efficiency of train ferry transportation.

## 1. Introduction

**Figure 1:**
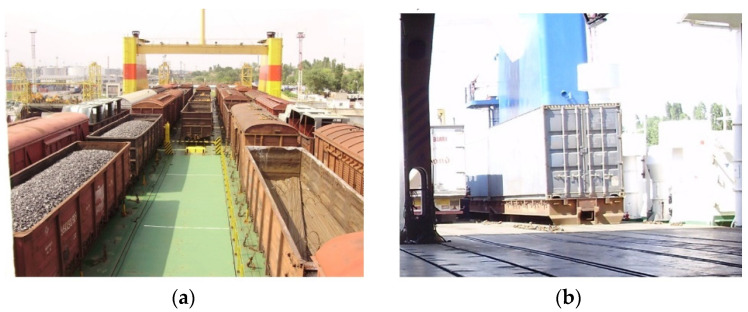
An example of rail vehicles transported by train ferries: (**a**) rail cars; (**b**) a container on a roll trailer.

The development of international freight transportation requires the introduction of combined interactions of different transport types. At present, the most widespread and prospective type is the transportation of rail cars by train ferries (Figure 1). For many countries, train ferry transportation is the access point to international transportation.

**Figure 2:**
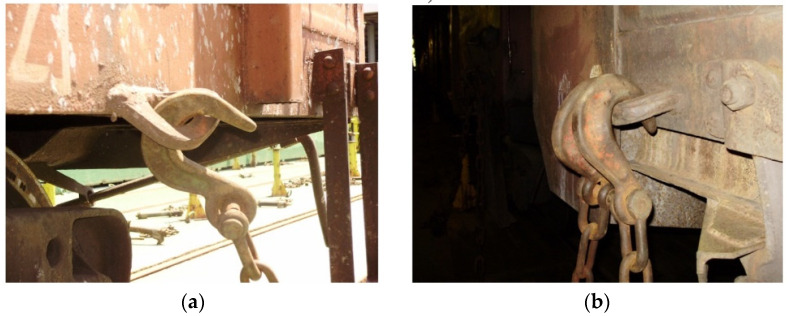
Damage in the structural components of open cars transported by train ferries: (**a**) solid-bottomed open car; (**b**) open car with hatch covers.

It should be noted that, apart from considerable advantages, this type of transportation has significant disadvantages because the vehicles transported are not intended for reliable interaction with train ferry decks. As well, the bearing structures of these vehicles are secured by the structural elements not intended for ferry transportation. Therefore, they bear considerable loads, exceeding the normative values established at the design stage (Figure 2).

This can undermine the stability of vehicles on the decks and cause accidents. Such accidents have already happened during the over-sea transportation of rail cars; they required off-schedule repairs and endangered ecological security regarding sea freight transportation. As an example, the Mercury–2 train ferry, which sank in the Caspian Sea, is considered. The vessel transported rail cars between Azerbaijan and Dagestan. It sank in October 2002, during a storm, due to the inappropriate securing of the rail cars. Unfortunately, this accident is not an isolated one. Similar accidents happened to train ferries Heraklion, Princess Ashika, Tõya Maru, and some others. Notably, such accidents with train ferries can have environmental consequences because they may transport various types of freight, including petroleum products, which require special safety measures during shipment.

Therefore, there is a need for research focused on vehicles’ loads while they are transported by sea, as well as on an adaption of railway vehicles for reliable interaction with train ferry decks.

## 2. Review of the Research and Literature

Many studies have been devoted to the loading and stability of sea vessels, for example [1,2,3,4]. However, research of the dynamics and better safety of railway vehicles during over-sea transportation have not been thoroughly studied.

The special properties of the modernized Sgnss freight railway wagons based on dynamic tests are described in [5]. The results of the numerical and experimental modelling of the load of a railway wagon are presented in this research. The analysis of the bearing structure strength of a modernized wagon is determined in [6]. The authors calculated the main strength values and strength of a railway vehicle’s main-bearing structure in terms of fatigue. They also considered the demands of modernization. It should be noted that these modernizations do not include the potential adaption of the railway vehicles’ main-bearing structures regarding their transportation by train ferries which worsens their operational efficiency during international transportation. Analysis of the structural features of a Zans wagon is considered in [7] where the authors presented the prospects of this rail car operation. The described research also includes the findings of the strength analysis of its bearing structure. In [8] the stress distribution of the main-bearing body of a Zans freight wagon car is also determined. The study describes the special features of the modified FE model (finite element model) of the analyzed structure of this wagon and the distribution of the maximal stresses. However, the load values for operation on main lines are taken into account for the design of the freight wagon structure. Thus, the study does not include the load occurring during train ferry transportation. The research focused on the optimization of the freight wagon’s main-bearing structure body is presented in [9]. Optimization was made for the open wagon body structure. The results of the research aimed at the analysis of the structure strength confirmed the efficiency of structural improvements for open freight wagons. The study [10] describes the special structural features of new-generation freight cars—BCNHL. The authors presented potential ways of improving the economic and technical values of freight wagons for better efficiency in terms of their operation. They also studied the structures of cars without components for their reliable fastening on train ferry decks. The modernization of freight cars is substantiated in study [11]. The aim of such modernization is better operational conditions for cars. However, such modernization does not ensure the safe transportation of freight wagons by train ferries. Study [12] describes the structural and dynamic analysis of a freight car. The simulation analyses were performed by means of the FEM (finite element method) and implemented by modern methods. The SolidWorks Simulation version 2015 software (Dassault Systèmes SOLIDWORKS Corp., Waltham, MA, USA) was used for setting up a 3-dimensional model of a freight wagon. Then, it was exported to the Ansys 2023 R1 software (Ansys, Inc., Canonsburg, PA, USA) and calculated using the finite element method. A system with many degrees of freedom was studied and compared with a system consisting of infinite degrees of freedom (so-called continuous structure). The recent task belongs to the large model’s category, i.e., the block Lanczos algorithm is used, which has high degrees of freedom (used by the PCG solver).

The dynamic load of the C70 railway wagon’s main-bearing structure for standard modes of operation corresponding to main lines was determined in scientific work [13]. The design of a model included the potential normative values of the loads. However, these studies did not take into account the determination of railway wagon dynamics regarding the operational conditions, i.e., it is transported by a ferry at the most adverse and unsafe load mode for its bearing structure. Studies [14,15] present the calculation of the dynamics and strength of the wagon’s main-bearing structures, which are transported by ferries. The authors suggest the techniques for their reliable fastening on the decks. At the same time, the authors think about improvement of the flat wagon to enhance the stability of containers when transported by sea. Thus, they propose special superstructures to be installed on the flat wagon. The authors also propose flexible connections that can be implemented by a viscous link to secure the cars on the deck in order to reduce the dynamic load of railway vehicles transported by sea. However, the load on these vehicles caused by a sea wave to the body of a train ferry was not included in the calculation. 

The work in [16] describes the method for determining the forces acting on railway wagons when transported by the sea. As an example, the authors conducted a study considering the hydrometeorological factors of the Caspian Sea. Based on the performed research, recommendations were formed aimed at ensuring the safe transportation of wagons by the sea. However, this approach did not take into account the influence of sea waves on the ferry hull. Also, the authors did not determine the stresses appeared in wagon bodies when transported by the sea.

The determination of the tensile forces due to the bulk cargo acting on the walls of an open wagon body when transported by railway ferry is covered in publication [17]. In this case, the authors used the classical Coulomb method, which was modified by Prof. Sinelnikova. The work also presents calculation schemes that can be taken into account for the assessment of the wagon bodies’ strength when transported by the sea. However, the authors did not perform the strength analyses of a wagon considering the proposed methodology.

The regulatory document [18] provides the rules for the safe transportation of freight wagons by the sea. Various schemes for securing the wagons on decks are described. The forces acting on the wagons’ bodies during slick-dry transportation have been analyzed. At the same time, the document indicates the magnitude of the acceleration applied to the wagons, with different course cutoffs applied to the ferry body. However, these forces apply to the single-deck ferry of the Petrovsk type, which was operated in the waters of the Azov Sea on the route Crimea–Caucasus. However, no studies were conducted regarding the water area of the Black Sea, through which the routes Ukraine–Bulgaria, Ukraine–Georgia, Ukraine–Turkey and others pass.

In [19], the features of the design of the fastening wagon methods on the decks of railway ferries are highlighted. The operation principle of these devices is examined. The work also provides a methodology of the strength calculation of fastening devices when interacting with wagon bodies. However, the authors did not pay attention to the issue of determining the wagon bodies’ strength when interacting with these devices.

Thus, there is a need to continue studies in the field and gather information on the safe transportation of rail cars by train ferries.

## 3. The Main Tasks and the Objective of the Research

The main objective of the presented research is to reveal the results of the research into the load on rail vehicles during train ferry transportation with consideration of the effect of a sea wave on the body of the ferry. This will contribute to the development of measures focused on railway vehicles’ safe transportation by the sea and better operational efficiency of train transportation by ferries. The defined objective can be achieved by means of the following main tasks:

- Modelling the dynamic load of vehicles when transported by train ferries with consideration of the effect of a sea wave on the ferry body;
- Calculating the basic strength parameters for the main-bearing body structure of an open railway wagon when transported by a train ferry;
- Performing an experimental study of the strength of the open wagon body during its transportation on a railway ferry;
- Offering measures for improvements in the open wagon main-bearing structure to ensure its reliable securing on the train ferry and;
- Studying the containers’ stability on the roll trailer located on the train ferry deck with consideration of the effect of a sea wave on the ferry body.

The solving procedure of these problems serves as the creation of the research methodology outlined in this presented research.

## 4. Creating a Computational Model

A mathematical model (1) has been formed to determine the dynamic load that acts on railway vehicles when transported on rail ferries by sea. This model was created using Lagrange’s equations of the second kind for non-conservative systems, i.e., with energy dissipation. In this case, kinetic energy and dissipation energy are taken into account as its components. These energies were determined using the classical formulas of Basic Ship Theory.

The load of the bearing structures of the vehicles on the train ferry deck under the impact load of the above-water projection regarding the dynamics was determined by means of mathematical model (1). The model included the rolling oscillations of the train ferry as the cause of the maximal load on the bearing structures of the vehicles located on it. It was also assumed that vehicles were rigidly secured on the deck and followed the displacement trajectory of the train ferry. The calculations were performed made for the deadweight carrying capacity of the rail ferry. 

**Figure 3:**
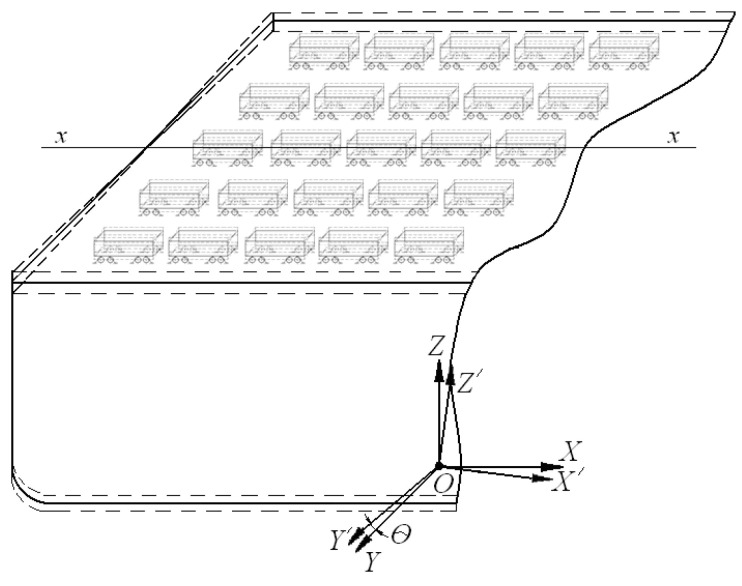
Diagram of movement of a train ferry laden with wagons during rolling.

The diagram of movement of the train ferry with railway vehicles during rolling is shown in Figure 3. The model included the disturbing impact of wind to the ferry’s above-water projection, as well as the impact load from a sea wave. The coordinate origin was in the mass center of the train ferry. Regarding the fact that the vessel made angular movements relative to the longitudinal axis, the vessel moment of inertia was taken as the inertia coefficient, i.e., the differential equations of motion were compiled with the classical approach used in mechanics.

The mathematical model describing the displacements of the train ferry under the impact interaction of a sea wave with its body has the form:

(1) $$\left[\frac{D}{12\cdot g}\cdot \left(B^{2}+4\cdot z_{g}^{2}\right)\right]\cdot \ddot{q}+\left(\Lambda_{\theta }\cdot \frac{B}{2}\right)\cdot \dot{q}=p^{\prime }\cdot \frac{h}{2}+P_{l}^{\prime }\cdot z\left(t\right),$$

where *q* = *θ*—generalized coordinate corresponding to the displacements of a train ferry with vehicles on its decks during rolling oscillations; *D*—weight displacement, kN; *B*—train ferry breadth, m; *h*—molded depth, m; Λ—oscillation resistance coefficient, kN∙s/m; *z_g_*—coordinate of the weight center of a train ferry, m; *p′*—wind load, kN; $P_{l}^{\prime }$—wave force, kN; *z*(*t*)—action law of the force disturbing the motion of a train ferry laden with freight wagons, m.

The law of the forces disturbing the motion of the train ferry loaded with vehicles on its decks was studied as a trochoidal motion. It has the following form:

(2) $$F\left(t\right)=a+R\cdot e^{k\cdot b}\cdot \sin\left(k\cdot a+\omega \cdot t\right)+b-R\cdot e^{k\cdot b}\cdot \cos\left(k\cdot a+\omega \cdot t\right),$$

where *R*—radius of the trajectory of the rotation of a particle, m; *a* and *b*—coordinates of the trajectory center of the motion of a particle in the horizontal and vertical directions with the coordinates *x* and *z* at a given time; *ω*—frequency of a sea wave, rad/s; *k*—wave number.

It should be said that the direction of the wave in relation to the hull of the railway ferry (heading angle *χ*) was taken into account in mathematical model (1). This is considered when setting the disturbance frequency *ω* dependent on the wavelength *λ*. When the motion of a vessel together with a wave at a specific value of χ is considered, the following known formulation can be written:

(3) $$\omega =\frac{2\cdot \pi \cdot \upsilon }{k_{\lambda }\cdot L\cdot \left|\cos\chi \right|},$$

where *υ* is the ferry speed, *k_λ_* is a coefficient that depends on the shape of the ferry’s contours, and *L* is the length of the ferry.

This approach is considered for specifying the disturbing influence in mathematical model (1).

The calculation is made for the Geroi Shipki class ferry that operates in the Black Sea and joins the Ukraine with Bulgaria and Georgia. This sea area was considered as an example because several strategically important ferry routes connecting the European countries pass through it. It is important to note that the proposed model can be applied to other sea areas and the railway ferries that operate within them. To do this, it is necessary to use the corresponding hydrometeorological characteristics of the sea water area in the model and the technical characteristics of the railway ferry for which the research is carried out.

**Figure 4:**
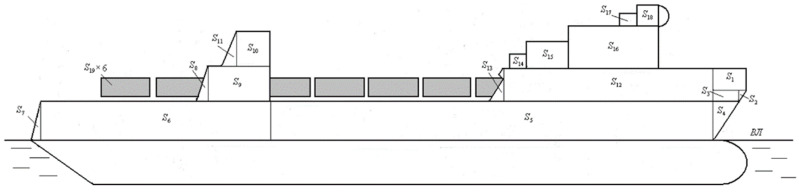
An above-water projection of the train ferry Geroi Shipki: *S*_1_–*S*_4_—lateral area on the stem; *S*_5_, *S*_6_—main lateral area; *S*_7_—lateral surface on the stern; *S*_8_–*S*_11_—lateral area in the boiler tubes; *S*_12_, *S*_13_—lateral area of the upper deck grillage; *S*_14_–*S*_16_—lateral area of the crew facilities; *S*_17_, *S*_18_—lateral area of the bridge; *S*_19_—lateral area of the sidewall of a rail car.

The wind pressure to the above-water projection of the train ferry was determined based on the album of drawings for the train ferry Geroi Shipki. The graphic reflection of its side projection was split into simple figures to determine its square (Figure 4).

The results from the performed calculation demonstrated that the square of the above-water projection was 2545.62 m^2^ and the wind pressure for the Black Sea was 1.47 kPa. This value of wind pressure was taken from the reference data for the Black Sea [17]. Thus, using the area of the above-water projection of the vessel and the wind pressure on it, it became possible to determine the wind force, which was taken into account in mathematical model (1). The impact of the sea wave was taken as 3 ton/m^2^. This is also a reference value and was taken from [20]. Here, the wave was considered as trochoidal and described by Equation (2). This waveform is described by Gerstner and Rankin. The wave’s length was 120 m and the period was 9 s.

It should be noted that the results of modelling the dynamics of the vessel with cars using model (1) and disregarding the wave impact were confirmed experimentally by one of the authors [18].

**Figure 5:**
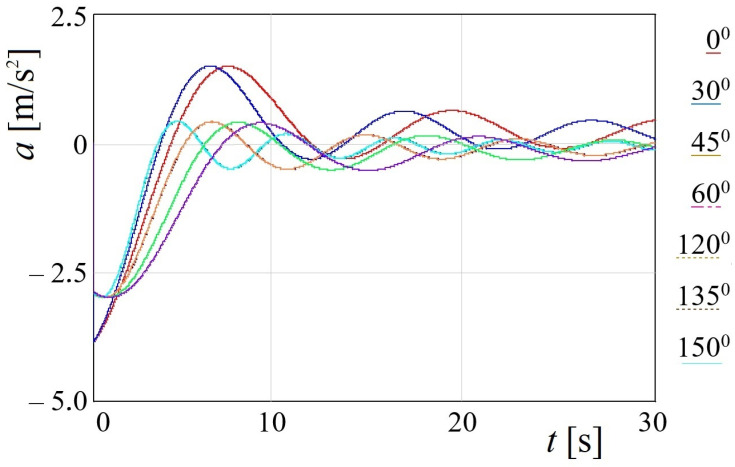
Accelerations to the wagon-bearing structure on the upper deck of the train ferry.

The mathematical model (1) was solved by means of the Runge–Kutta method [21]. The initial conditions, i.e., displacements and velocities, were taken close to zero [22,23,24]. It should be noted that the verification of the solution correctness of this equation is confirmed by the method of varying arbitrary constants. The calculation included different sea wave angles regarding the train ferry body. The results of the calculation are shown in the form of graphs in Figure 5.

Figure 5 demonstrates that the maximum acceleration corresponds to a wave angle to the body of 0° and equals 3.6 m/s^2^. In this case, each colored line shown to the right of the graph in Figure 5 corresponds to a certain wave angle. The value of acceleration obtained was 37% higher than the operational value for the car on the main lines when the motion was estimated as admissible [25,26]. It is worth noting that this acceleration value was calculated for the car that was the farthest from the bulwark and located on the upper deck of the vessel. It should also be noted that mathematical model (1) was verified by means of the F-test, as presented in [27]. However, that model did not consider the influence of the wave on the vessel hull.

The acceleration calculated was taken for the calculation of the strength of an open wagon main-bearing structure when it was secured on the deck of a train ferry with chain binders.

The strength analyses of the open wagon main-bearing structure during its interaction with the lashing devices were studied by means of a three-dimensional model, which was set-up in the SolidWorks Simulation version 2015 software. This calculation was carried out not only to assess the strength of the body when it is transported on a railway ferry, but also from the maritime transport safety point of view. Violation of the wagon stability on the deck is fraught with the loss of the railway ferry’s stability.

The model included only those elements which were rigidly connected to each other (welded or riveted). Because hatch covers were not connected to the body frame by means of rigid elements, but they were connected to the main-bearing structure by means of joints, which transfer the load to the frame elements. Therefore, they could be not considered in the model.

**Figure 6:**
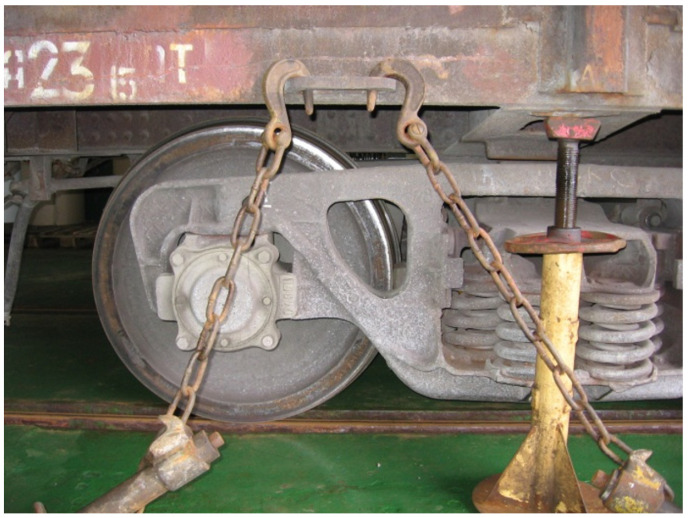
Securing of the open wagon on the deck.

The created model has not included the longitudinal loads acting to the open wagon body through the coupler because they have been restricted by the fixed fitting installed at the end of a car batch and with the braking shoes. The fastening of an open car was simulated by means of additional joints in the locations where the bearing structure is placed on the bogie’s center pivots, slides, and jacks. The most unreliable securing of the open wagon on the deck was included in the calculation (Figure 6).

**Figure 7:**
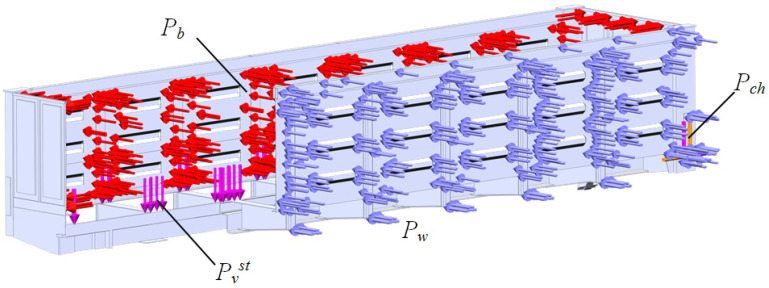
A scheme of the loads defined on the open wagon main-bearing body structure.

A scheme of the load acting on the open wagon main-bearing body structure is shown in Figure 7. This scheme includes the following loads: static load in the vertical direction *P_vst_*, pressure due to transported bulk cargo *P_b_*, dynamic, and wind *P_w_*, and loads to the wagon body through the chain holders *P_ch_*. The dynamic load from chain binders and the pressure due to the bulk cargo to the bearing structure of the open wagon was included. The design diagram is shown in section for better visualization of the loads applied. When the wagon body is tilted, four ties on one side of the car absorb not only the preload (≈50 kN), but also the dynamic load, which was determined considering the result obtained from the mathematical model (1) acceleration. On the opposite side of the wagon, it was considered that the ties are loaded only by the preload.

**Figure 8:**
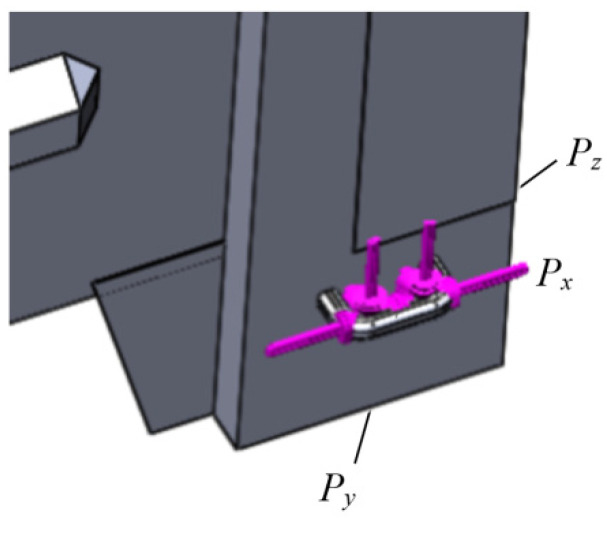
Diagram of the load acting from the chain holder to the towing shackle.

As the chain holders are located spatially, it leads to the decomposition of the load, which acts on the open wagon main-bearing structure body (Figure 8).

These calculations were made using the FEM. The isoparametrical tetrahedrons were defined for the created FE model [28,29,30,31]. The number of elements of the FE mesh was 494,440, the number of units was 160,329, the maximum element size was defined to the value of 80 mm, the minimum element size was chosen to the value of 16 mm, the maximum element side ratio was determined to the value of 550.85, the percentage of the created elements with a side ratio of less than three was evaluated to the value of 27.3, and more than ten reached the value of 29.3. The element gain ratio in the mesh was 1.7. The circle included 9 numbers of the elements.

Coal was chosen as the bulk cargo as it is one of the most widespread types of freight transported in open cars by train ferries. The pressure from the bulk cargo to the walls of an open car was determined by the method based on Coulomb’s law. The value of acceleration to the car body was also included in the calculation. Thus, the pressure from the bulk cargo was determined by formula [20]:

(4) $$p=\gamma^{\prime }\cdot h^{\prime }\cdot \alpha \cdot g\pm F_{ad},$$

(5) $$\alpha =\frac{\cos^{2}\left(\rho^{\prime }+\alpha^{\prime }\right)}{{\left[1+\sqrt{\frac{\sin\rho^{\prime }\cdot \sin\left(\rho^{\prime }\pm \alpha^{\prime }\right)}{\cos\alpha^{\prime }}}\right]}^{2}\cdot \cos\alpha^{\prime }},$$

where *h*′ represents the height of the open wagon body; *γ*′ is the volume weight of the freight; *ρ*′—angle of internal friction; *α*′ represents the open wagon rolling angle relative to the longitudinal axle; *F_ad_* is the additional pressure caused by the component of the bulk cargo dynamics due to the oscillations of a train ferry.

**Figure 9:**
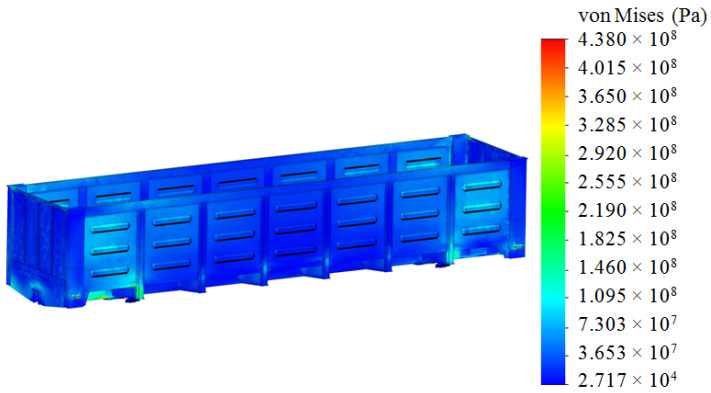
A distribution of stresses in the open wagon body (side view).

**Figure 10:**
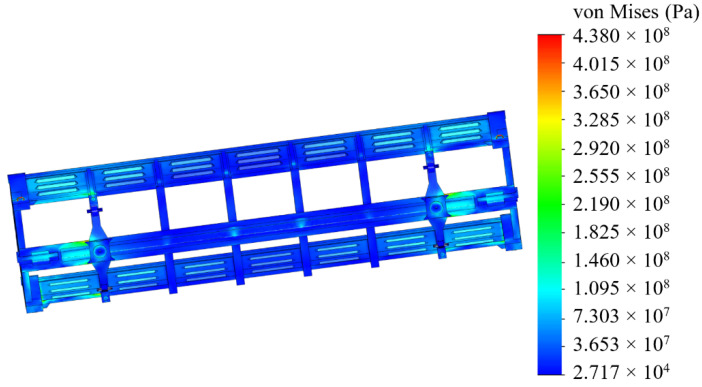
A distribution of stresses in the open wagon body (bottom view).

**Figure 11:**
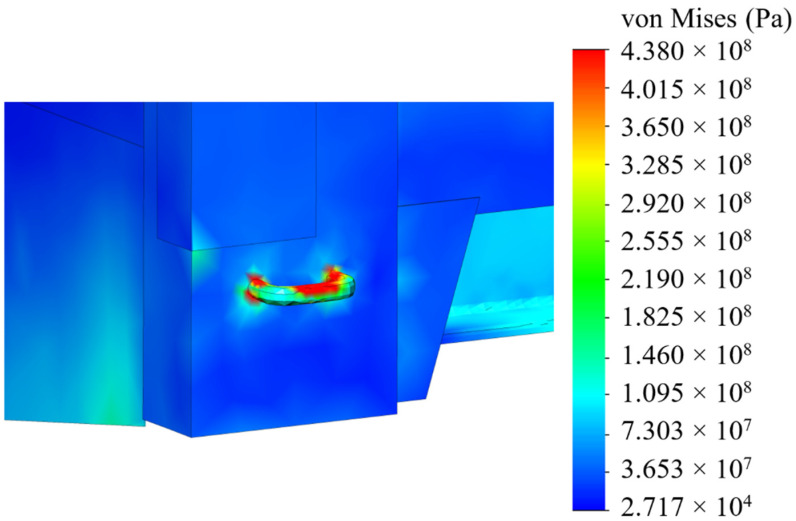
A distribution of stresses in the most loaded zone of the structure.

The analyses of the strength of an open wagon have been carried out by means of the FEM and the SolidWorks CosmosWorks Simulation version 2015 software (SolidWorks Corporation, Concord, MA, USA) was used for it. The results of the performed analyses are depicted in Figure 9, Figure 10 and Figure 11.

The results of the performed simulations demonstrated that the maximal equivalent stresses of the analyzed main-bearing structure of the open wagon body achieved the value of 438 MPa, concentrated in the towing shackle. Thus, they exceeded the allowable values by 21% [25,26]. The maximal deflections of the structure occurred in the sidewall structure of the open wagon body and the achieved value equaled 15 mm. Thus, this scheme to secure the considered open wagon on the train ferry deck should not be used. Therefore, the need to adapt the main-bearing structure of the open wagon for transportation by train ferries has appeared.

**Figure 12:**
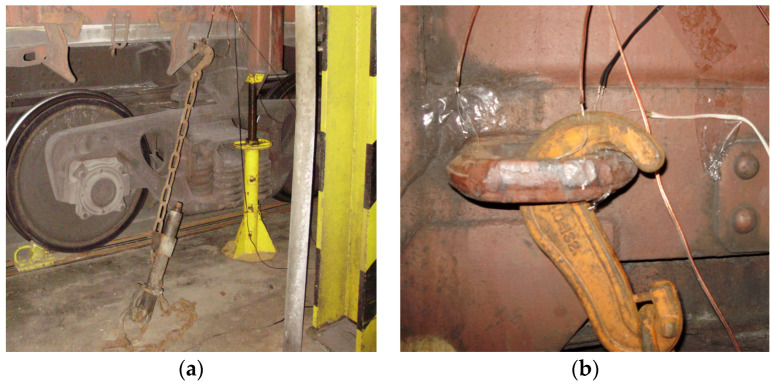
A scheme of fastening the researched open car on the deck: (**a**) Fastening the chain hook to the locking sector of the hatch cover; (**b**) Fastening the chain hook to the bracket for pulling up the wagon during shunting operations.

The full-scale tests were carried out on the deck of the railway ferry Geroi Shipki during the Black Sea voyage. These tests have been performed in order to investigate the strength of the open wagon body by means of experimental studies (Figure 12a) [20]. In this case, the method of electrical strain gauging was used, which has found wide application in mechanical engineering to determine the strength of structures under operating loading conditions. The study was carried out on a universal open wagon with a carrying capacity of 69 tons. The vehicle was placed on the main deck. Fastening of the open wagon on the deck is shown in Figure 12b. During the research, the sea wave was 4–5 points, the wave course angle regarding to the hull of the railway ferry was set at a value of 60° to 80°, and the wind speed and direction were 6 to 10 m/s and 60° to 80°, respectively. The railway ferry roll angle was about 5°.

The largest value of trim of the railway ferry was recorded on the approach to Cape Sarich and it was about 0.7 m on the stern, which corresponds to an angular displacement of less than 1°.

**Figure 13:**
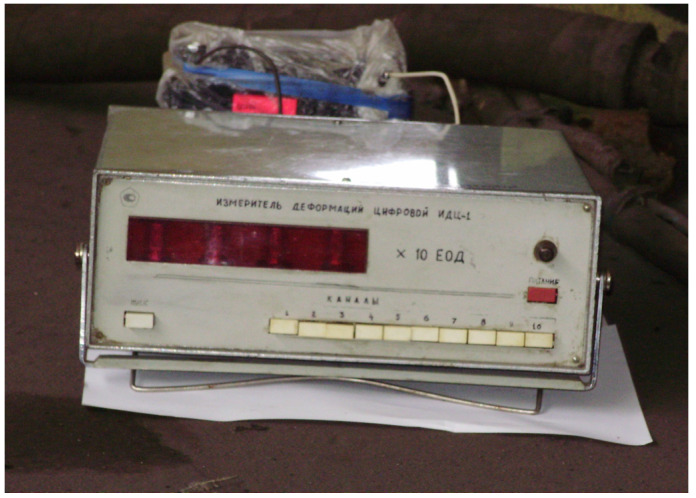
A device for measurement of deformation gauge of construction and machine-building structures of VDC—1.

**Figure 14:**
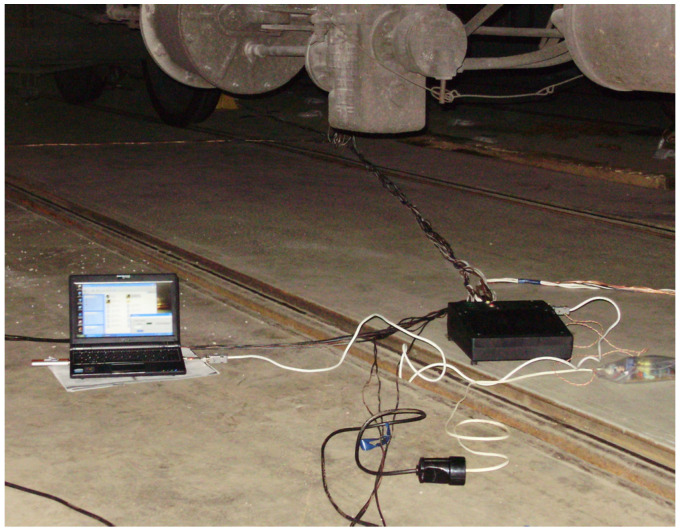
Connecting the computer to the strain gauges.

In order to determine the forces in the locations of interaction of the open wagon body with chain hooks, the following technical means were used:

1. A device for measurement of deformation gauges of construction and machine-building structures of VDC—1 (Figure 13); VDC—1 removes and converts the received information into values of physical quantities with subsequent display of information about the measured readings of the object, in this case—deformation. The device has an input polling time of no more than 1 s and one input (channel). The DC supply voltage range is 10.5–30 V. The maximum power consumption of the device is no more than 2 VA. The kit includes removable terminal blocks for easy installation and dismantling of the device. VDC—1 has the ability to receive information from strain gauges with different technical characteristics;
2. A universal tester;
3. Wire tension resistors with a base of 20 mm and resistance of 124 Ohms;
4. A set of mounting wires for connecting the strain gauge;
5. A computer for the purpose of storing the data registered during the study (Figure 14).

The method of electric voltage measurement was applied for an experimental study of forces in the locations of interaction of an open wagon body with chain hooks.

The installation points of strain gauges on the open wagon body were chosen based on cases of real schemes of fastening regarding the common locations on the deck, which are oriented relatively to the regular point on the deck in the areas of interaction of the body with the chain hooks. The half-bridge circuits were made to connect the sets of tensor resistors in the research areas.

**Figure 15:**
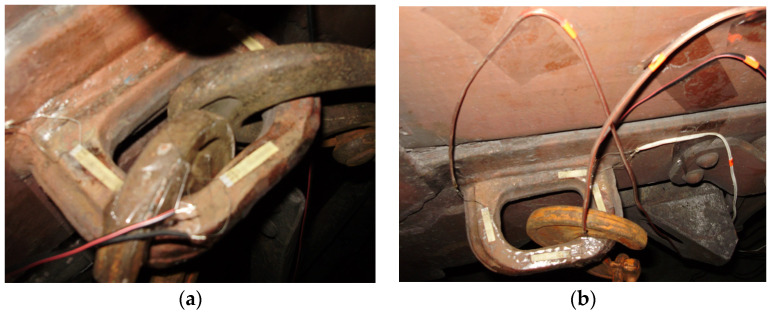
A placement of strain gauges on the body: (**a**) A tow bracket with two chains attached; (**b**) A tow bracket with one chain attached.

The tensor resistors were attached to the surface of the studied areas of the open car body by means of thermo-reactive glue based on Cyacrine. Before that, the surface of the open car body under sensors were cleaned to a matte finish and degreased. The placement of the strain gauges on individual elements of the open wagon body secured relative to the decks is shown in Figure 15.

**Figure 16:**
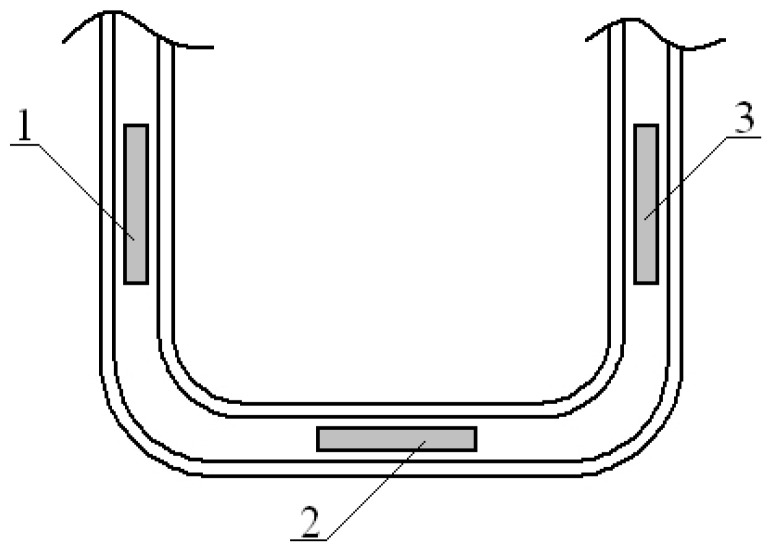
A scheme of placements of strain gauges placed on the tow bracket for pulling up the open car during shunting.

**Figure 17:**
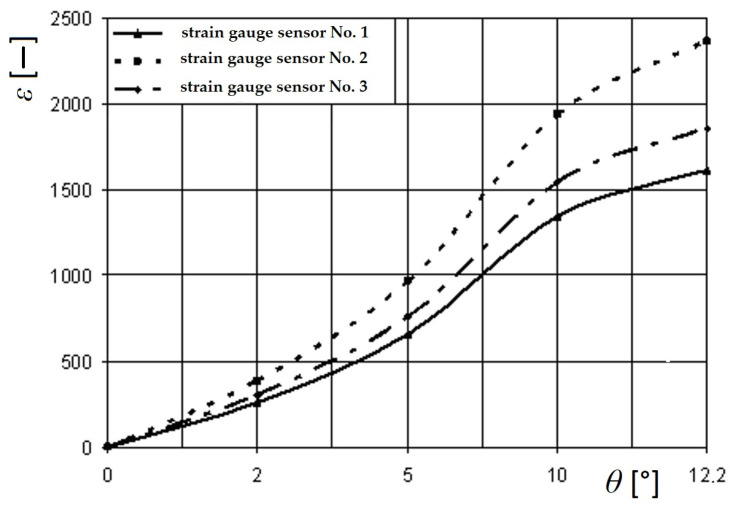
Dependence of the relative deformations on the roll angle in the locations of the strain gauges’ placements on the tow bracket used for pulling up the open car during shunting operations.

A scheme of placements of strain gauges placed on the tow bracket, used for pulling up the open car during shunting, is depicted in Figure 16. The readings of the strain gauges were recorded automatically. In this case, a special software was used. The readings taken from the sensors were converted into a table of values in Excel 2021 (Microsoft 365) software (Microsoft Corporation, Redmond, Washington, DC, USA). Based on the obtained sample, an analysis of the test results was carried out. The greatest values of relative deformations in the open car body were recorded by the set of strain gauges located on the closing sectors of hatch covers and the tow bracket for pulling up the open car. It was within the assumption that two chain hooks were attached to it, which amounted to 865 and 996 units, corresponding to the values of 178 MPa and 203.4 MPa, respectively (Figure 17). 

**Figure 18:**
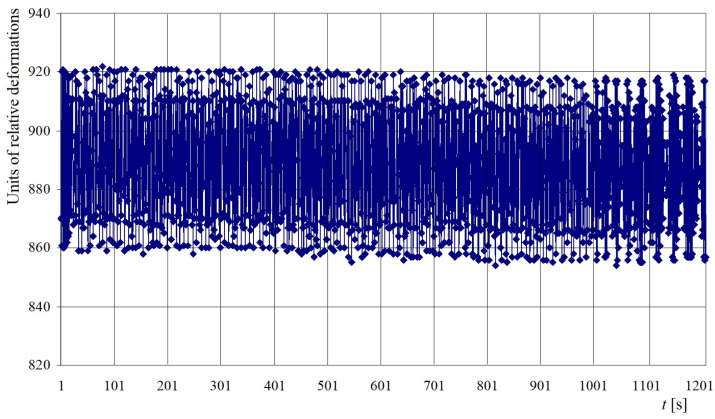
Time dependence of relative deformations in the location of the attachment of the open wagon body regarding the deck.

Figure 18 shows the graphical dependence of the relative deformations in the area of attachment of the open wagon body regarding the deck (tow bracket) on the parameters of the sea wave (length, height, frequency, etc.).

From this dependence, it can be concluded that the relative deformations in the attachment zones of the open wagon body regarding the deck have stochastic characteristics and depend directly on the parameters of the sea wave.

The results of the performed analysis, which was focused on the investigation of the maximal values of the strain gauge from experiments, are listed in Table 1. 

To assess measurement accuracy, their errors were calculated, defined as the sum of systematic Δ*ε_c_* and random Δ*ε_b_* measurement errors [32]:

(6) $$\Delta \varepsilon =\Delta \varepsilon_{c}+\Delta \varepsilon_{b},$$

For a standard beam of equal resistance, Δ*ε_c_* is 4%. The random measurement error was determined according to the method described in [32] and amounted to a value of 4.1%. The total measurement error was about 8%.

The Fisher’s criterion (F-test) was carried out to verify the adequacy of the developed model, which served for evaluation of the strength of the open wagon body. At the same time, the schemes of its fastening regarding the deck have been considered. As it is known, the test’s statistics one way or another comes down to the ratio of sample variances. In this case, two samples were created—theoretical and experimental. The number of sample elements was determined using Student’s *t*-test. The roll angle of the railway ferry was considered as a variation parameter. Next, using the methods of mathematical statistics, the value of the variances of adequacy and reproducibility was determined. The ratio of these two variances allows to determine the theoretical value of the criterion, which is compared with the tabulated value.

The reached actual value of the Fisher’s criterion *F_p_* = 5.11 is lower than the value introduced in the table, namely the value of the F-test, *F_t_* = 5.41. It is based on the performed calculations with the reproducibility variance *S_y_* = 6.94 and the adequacy variant *S_ad_* = 35.42, which is lower than the table value of the criterion. Thus, the adequacy hypothesis is not denied. The approximation error value was about 2%.

**Figure 19:**
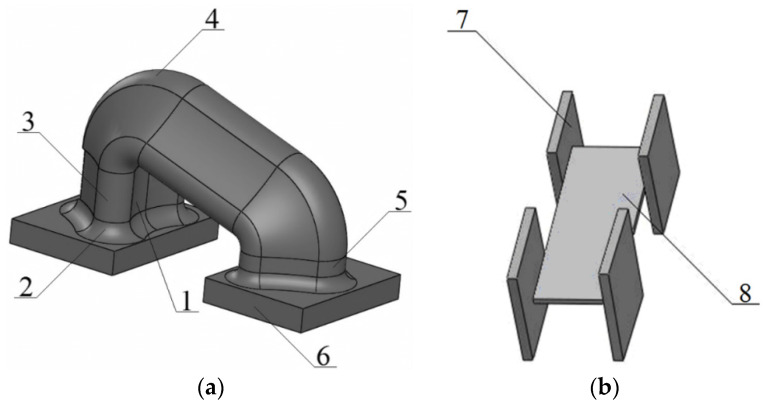
Unit for securing the wagon on the train ferry deck: (**a**) Components; (**b**) Reinforcing diaphragm.

The research team has proposed a device for securing the open wagons on a ferry to ensure its safe transport. This proposed unit consists of several parts. These include: the bottom part, together with a hook guide (item 1) (Figure 19). This bottom part entirely follows the geometry configuration between the hook of the chain holder, and it is intended to interact with the fastening unit. The purpose of a radial lug (item 2) is to reduce the load, which is concentrated in the area of the interaction of the unit and the support part, i.e., the cylindrical part (item 3). It provides a sufficient interaction of the hook with the unit. The upper part of the body includes the prismatic part (item 4) which is intended for connection between the working part of the unit and the additional part with technological reinforcement (item 5). The support parts (item 6) of the unit are intended securing it on the bolster beam of the wagon. The stiffness of the bolster in the units can be reinforced with diagrams consisting of pads (item 7) and a connective pad (item 8).

The strength analysis of the main-bearing structure of the freight wagon was calculated with consideration of the solutions proposed. The design diagram was similar to that given in Figure 7, except for the load to the main-bearing structure of the freight wagon through the chain binders, which was applied to the cylindrical parts of the unit.

**Figure 20:**
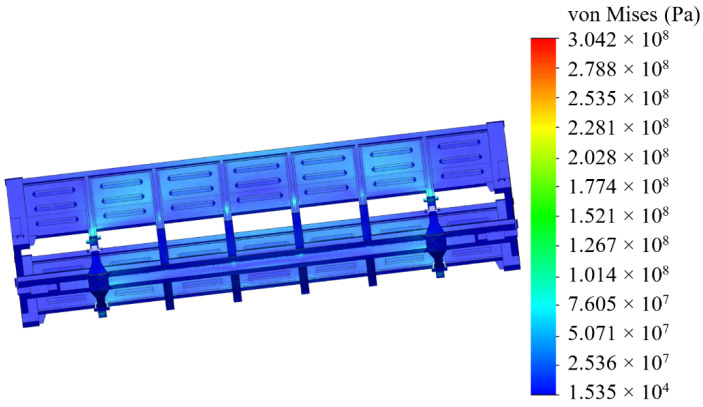
A distribution of stresses in the open wagon body with the lashing scheme developed.

**Figure 21:**
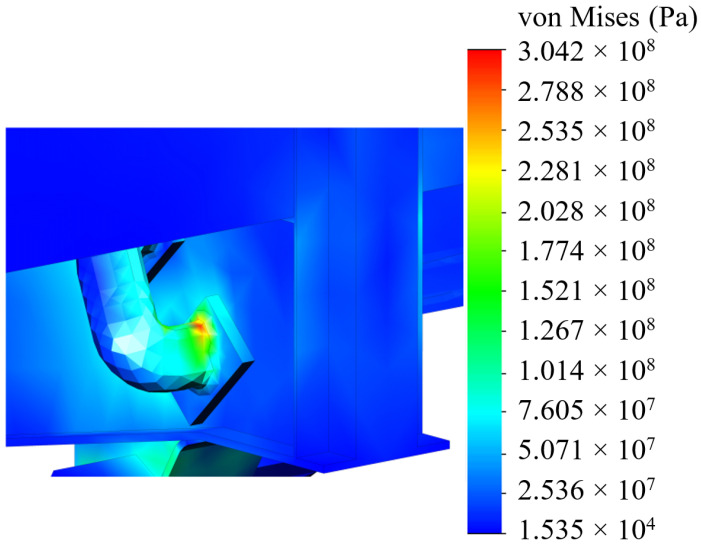
Detail of the stress distribution of the open wagon body in the location of the maximum stresses for the developed lashing scheme.

Figure 20 and Figure 21 depict the results of the performed simulation calculation. The maximum equivalent stresses were concentrated in the radial lug of the unit and amounted to 304 MPa. Thus, the permissible value [25,26] was not exceeded and was lower by 30% than those in the typical fastening scheme. Therefore, the sufficient strength of the open wagon main-bearing structure was preserved.

**Figure 22:**
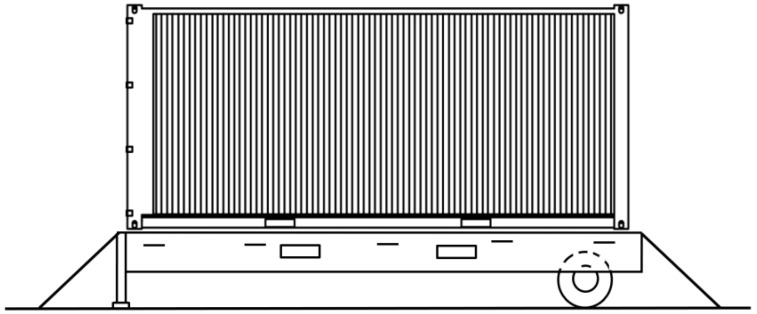
Fastening of a roll trailer on a train ferry deck.

The obtained values of acceleration were also considered in the investigation of the container on the roll trailer when transported on the train ferry. The lashing scheme for a roll trailer on a train ferry deck is presented in Figure 22.

**Figure 23:**
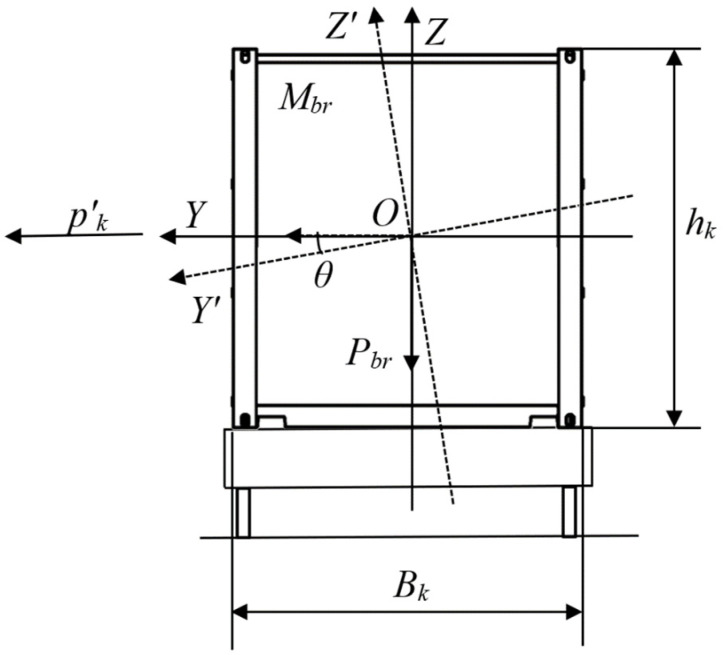
Diagram of a considered container.

Figure 23 shows the results of the performed calculations.

The equilibrium condition has the form:

(7) $$k_{S}=\frac{M_{rest}}{M_{dist}}=\frac{P_{br}\cdot \cos\theta \cdot \frac{B_{K}}{2}+n_{f}\cdot \left[M_{br}\cdot \left(g\cdot \sin\theta +{\ddot{\theta }}_{k}\right)\right]\cdot \frac{h_{f}}{2}}{p_{k}^{\prime }\cdot \frac{h_{K}}{2}+M_{br}\cdot \left(g\cdot \sin\theta +{\ddot{\theta }}_{k}\right)\cdot \frac{h_{K}}{2}}\geq 1,$$

where *M_rest_*—restoring moment value, Nm; *M_dist_*—disturbing moment value, Nm; *M_br_*—gross mass of a container, kg; *P_br_*—gross weight of a container, kg; *B_k_*—breadth of a container, m; *n_f_*—number of container fittings to support a container during the angular deflections considered relatively to the axle in the longitudinal direction; *h_f_*—height of the container fitting, m; *θ*—roll angle, °; ${\ddot{\theta }}_{k}$—acceleration regarding the standard location of the container on the deck, m/s; *h_k_*—container height, m; $p_{k}^{\prime }$—wind pressure acting on the container, Pa.

**Figure 24:**
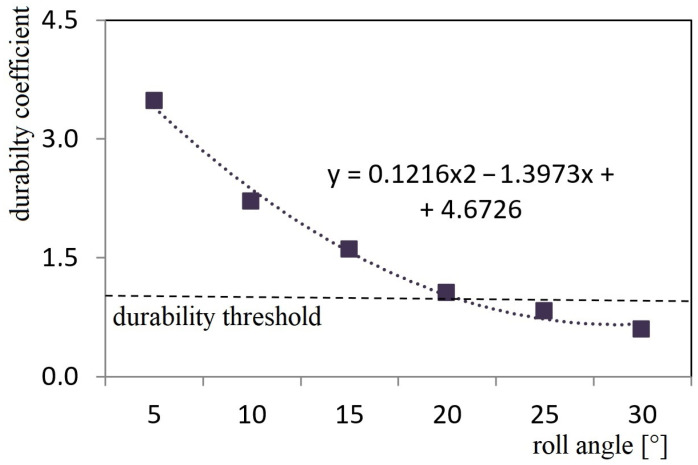
Dependency of the coefficient stability of a considered container located on a roll trailer on the rolling angle of a train ferry.

During their research, the authors obtained the dependency of the stability coefficient of a container located on a roll trailer on the roll angle of a train ferry (Figure 24).

The research showed that the stability of the considered container on the roll trailer in the standard interaction diagram (fixed fitting—container fitting) was provided at rolling angles of up to 20°. Significantly, the calculations did not include the potential movement of goods loaded in the container, as well as the asymmetry of its location on the roll trailer, i.e., the calculation was carried out for a container inclined during rolling. These factors, which may impact the stability of the container, will be included in future research.

## 5. Discussion of the Results of the Research

The presented research aims to determine the dynamic load on the bearing structures of rail vehicles during their train ferry transportation with consideration of the effect of a sea wave to the body. The calculation was based on mathematical model (1). The model included rolling oscillations of the train ferry. Further, it was considered that the vehicles were fastened rigidly to the deck and their displacements followed those of the train ferry. It was found that, under the hydro-meteorological conditions of the Black Sea, the acceleration to the proper location of a vehicle was 3.1 m/s^2^ (Figure 5). The value of acceleration was 37% higher than the operational acceleration accepted for the car moving on the main line if its motion was estimated as admissible. 

The calculated value of acceleration was input to the analysis of the strength of the open wagon main-bearing structure. The open wagon was chosen because it represents the most widespread type of wagons transported by sea. The restriction of the designed model was its quasi-static nature. It should also be noted that the limitation of the developed model intended to be applied for strength analysis of the wagon main-bearing structure consists of its absence of welded joints, i.e., the model was considered as monolithic. Thus, it was found that the application of the standard scheme for securing the railway wagon on the deck is not admissible. 

To confirm the theoretical results, full-scale experimental tests, which focused on the strength analyses of the open wagon body when transported by a rail ferry, were performed. For these purposes, the method of electrical strain gauging was used. The points of installation of the strain gauges on the wagon body were determined based on the actual scheme for securing the car to the deck, depicted in Figure 15. Based on the obtained experimental results, verification was carried out using Fisher’s criterion (F-test). It was concluded that the hypothesis of adequacy will not be rejected.

The authors suggested the structural fastening unit, which is intended to secure a freight wagon on a ferry deck (Figure 12). The achieved results of the strength analyses led to the finding that the maximal value of the equivalent stresses was 304 MPa (Figure 14). Thus, these values do not exceed the permissible values.

The accelerations obtained from the derived mathematical model (1) were included for analysis of the stability state of a container placed on a roll trailer and transported at the same time by a train ferry. It was found that the degree of stability of a considered container placed on a roll trailer in the standard scheme of interaction was preserved at rolling angles of up to 20° (Figure 24). It is important to note that the analysis of the regulatory documents focused on securing railway vehicles on railway ferries [33,34,35,36] allow to conclude that they are identical. Therefore, the methodology proposed in the research can be applied to other types of railway ferries.

A limitation of this study is that it is valid to determine the load of vehicles during the cruise speed of a railway ferry. In the future, it is necessary to consider the presence of several degrees of freedom of the railway ferry. Also requiring attention is the issue of taking into account the stochasticity of sea waves and their influence on the fatigue strength of the wagon body, with the proposed option of fastening on the deck also taken into account. This will be considered in subsequent studies by the authors’ team.

It is important to note that a preliminary calculation of the economic effect of using special units for securing wagons on the deck allowed to conclude the feasibility of this solution. The cost of equipping the wagon with such units was about 600 euros. The economic effect is achieved by reducing costs for unscheduled repairs of wagons.

A potential problem for the implementation of the proposed wagon fastening units to consider is the installation of additional diaphragms in the pivot beam. Fastening of these units is possible using overlap welding with a double seam. At the preliminary stage, work is underway on this issue. It is also necessary to consider the presence of corrosive wear of vertical sheets, which entails a decrease in the rigidity of the pivot beam when accepting operational loads.

The conducted research can be used to develop measures for the safe transportation of railway vehicles by sea and to improve the operational efficiency of train ferry transport [29].

## 6. Conclusions

1. The presented research focuses on modelling the dynamic loads of railway vehicles during their train ferry transportation with consideration of the effect of sea waves to its body. The maximum acceleration, which occurs at a wave angle regarding the train ferry body of 0°, is calculated at the value of 3.6 m/s^2^.
2. The study presents the determination of the main strength characteristics of the main-bearing structure of the open freight wagon when transported by train ferries. The maximum equivalent stresses in the open freight wagon body are up to the value of 438 MPa and are concentrated in the towing shackle; thus, they exceed the admissible values by 21%. The maximum displacements occur in the sidewall of the open car body, equal to 15 mm. Thus, this scheme to secure the railway wagon on the deck of a train ferry is inadmissible.
3. Additionally, the strength analysis of the open wagon body mounted on the railway ferry deck was carried out by means of experimental tests. For these purposes, the method of electric strain measurement was applied. In this case, strain gauges with a base of 25 mm and resistance of 124 Ohms were used. Based on the experimental results obtained, verification was carried out. The experimental tests have revealed that the Fisher’s criterion actual value was *F_p_* = 5.11, which is within reproducibility variance *S_y_* = 6.94 and adequacy variance *S_ad_* = 35.42. This value is lower than the value of the criterion *F_t_* = 5.41 introduced in the table. Thereof, the hypothesis of adequacy is not denied. The approximation error was about 2%.
4. Based on the performed research, the measures of improving the main-bearing structure of the open freight wagon for providing its secure transport on the train ferry were suggested. Particularly, it includes the mounting of special fastening units on the bolsters of the cars. The results of performed calculations demonstrate that the maximal values of the equivalent stresses are concentrated in the radial lug of the unit and amounted to a value of 304 MPa. Thus, these values have not exceeded the permissible values. At the same time, the achieved values are lower by 30% than those in the standard fastening scheme. Thereof, the strength of the one wagon main-bearing structure is preserved.
5. The conducted research was also focused on the investigation of the stability of a container, which is placed on a roll trailer located on the train ferry deck. The impact of a sea wave to the ferry body is considered. It is found that the stability of the investigated container placed on the roll trailer, with consideration of the standard interaction scheme, is ensured at rolling angles up to a value of 20°.

## 7. Patents

Viznyak R.I., Lovska A.O. The node of the load-bearing structure of the car body for its fixation relative to the deck of the railway-ferry vessel: pat. 108214 Ukraine: (2015.01) B60P 7/08 (2006.01) B60P 7/135 (2006.01) B60P 3/06 (2006.01) B61F 1/12 (2006.01); application 21.05.12; publ. 10.04.15. Bull. #7. (in Ukrainian).

## Figures and Tables

**Table 1:** Results of statistical processing of the measurements.

*θ* [°]	Measurement Series	Strain Gauge No.	Average Value	Minimal Value	Maximal Value	Dispersion	Mean Square Deviation
5	3	1	512	464	514	1.2	1.1
	3	2	920.5	844	921	0.5	0.7
	7	3	612	585	641	0.68	0.82

## Data Availability

Data are contained within the article.
